# Rationale and design of the Kanyini guidelines adherence with the polypill (Kanyini-GAP) study: a randomised controlled trial of a polypill-based strategy amongst Indigenous and non Indigenous people at high cardiovascular risk

**DOI:** 10.1186/1471-2458-10-458

**Published:** 2010-08-05

**Authors:** Hueiming Liu, Anushka Patel, Alex Brown, Sandra Eades, Noel Hayman, Stephen Jan, Ian Ring, Greg Stewart, Andrew Tonkin, Tarun Weeramanthri, Vicki Wade, Anthony Rodgers, Tim Usherwood, Bruce Neal, David Peiris, Hugh Burke, Christopher Reid, Alan Cass

**Affiliations:** 1 The George Institute for International Health, PO Box M201, Missenden Road, NSW 2050, Australia; 2 Baker IDI Centre for Indigenous Vascular & Diabetes Research PO Box 1294, Alice Springs, Northern Territory 0871, Australia; 3 Baker IDI Heart and Diabetes Institute, PO Box 6492, St Kilda Road Central, Victoria 8008, Australia; 4 Inala Indigenous Health Service, 64 Wirraway Pde, INALA, QLD, 4077, Australia; 5 University of Wollongong, Northfields Ave, Wollongong NSW 2500, Australia; 6 Sydney South West Area Health Service, Eastern Campus Of Liverpool Hospital Elizabeth St, Liverpool NSW 2170, Australia; 7 Monash University, Victoria 3800 Australia; 8 WA Health, Level 3, B Block, 189 Royal Street, East Perth WA 6004, Australia; 9 The University of Sydney, PO Box 154, Westmead, NSW 2145. Australia; 10 Maari Ma Aboriginal Corporation, 443 Argent Street, PO Box 339 Broken Hill, NSW, 2880, Australia

## Abstract

**Background:**

The Kanyini Guidelines Adherence with the Polypill (Kanyini-GAP) Study aims to examine whether a polypill-based strategy (using a single capsule containing aspirin, a statin and two blood pressure-lowering agents) amongst Indigenous and non-Indigenous people at high risk of experiencing a cardiovascular event will improve adherence to guideline-indicated therapies, and lower blood pressure and cholesterol levels.

**Methods/Design:**

The study is an open, randomised, controlled, multi-centre trial involving 1000 participants at high risk of cardiovascular events recruited from mainstream general practices and Aboriginal Medical Services, followed for an average of 18 months. The participants will be randomised to one of two versions of the polypill, the version chosen by the treating health professional according to clinical features of the patient, or to usual care. The primary study outcomes will be changes, from baseline measures, in serum cholesterol and systolic blood pressure and self-reported current use of aspirin, a statin and at least two blood pressure lowering agents. Secondary study outcomes include cardiovascular events, renal outcomes, self-reported barriers to indicated therapy, prescription of indicated therapy, occurrence of serious adverse events and changes in quality-of-life. The trial will be supplemented by formal economic and process evaluations.

**Discussion:**

The Kanyini-GAP trial will provide new evidence as to whether or not a polypill-based strategy improves adherence to effective cardiovascular medications amongst individuals in whom these treatments are indicated.

**Trial Registration:**

This trial is registered with the Australian New Zealand Clinical Trial Registry ACTRN126080005833347.

## Background

Socioeconomically disadvantaged populations are at high risk of chronic vascular disease. In Australia, this is particularly the case for Indigenous peoples, amongst whom more than one third of the total disease burden is due to cardiovascular disease (CVD), chronic kidney disease (CKD) and diabetes[1]. Six risk factors (tobacco, overweight, high cholesterol, physical inactivity, high blood pressure, and low fruit and vegetable intake) explain the majority of this burden[1]. Current national guidelines for the prevention of cardiovascular events in people with established athero-thrombotic vascular disease, or at high risk of these events, recommend - unless contraindicated - aspirin, Angiotensin Converting Enzyme (ACE) inhibitors and statin therapy[2-5].

The George Institute for International Health and the Kanyini Vascular Collaboration (KVC) have recently completed three cross-sectional studies of CVD risk management in Australian general practice and in Aboriginal Medical Services (AMS) settings[6-8]. The KVC Audit showed that, amongst a random sample of 1165 Indigenous adults, 40% of patients with established CVD had not been prescribed the combination of blood pressure (BP) lowering medicines, statins and antiplatelet agents and that 56% of high risk individuals without CVD had not been prescribed BP medicines and statins[7]. Actual adherence is likely to be even lower. Similar screening and treatment gaps were found for predominantly non-Indigenous adults in mainstream general practices[8] and in other Australian and international studies[9-14].

The reasons for the current evidence-practice gaps are likely to be complex. Barriers to adopting guideline recommendations by doctors might include lack of time, a confusing multiplicity of guidelines, lack of awareness of guidelines, and insufficient resources to implement recommendations[15]. Low adherence to medication is a well-documented barrier to the continued prevention and treatment of chronic diseases[16-21]. Non-adherence is associated with taking multiple medicines with complex dosing regimens, inadequacy of knowledge about the medications and depression[16,17,20-22]. As cost is an important contributing factor, patients adopt strategies to reduce costs - including not filling prescriptions and delaying or omitting doses[20,23]. Aboriginal people's inequitable access to medicines subsidised through the Pharmaceutical Benefits Scheme has been clearly demonstrated[24].

While the use of a 'polypill' for primary prevention in a population-based approach among people at low risk remains controversial[25], the potential role of fixed-dose combination therapy in secondary prevention amongst people suffering from CVD, or who are at high risk of such events, has gained wider acceptance[19,26,27]. A systematic review of randomised trials comparing the effects of combined packaging of pills or fixed-dose combination pills with access to the same medications presented as separate pills, demonstrated improvements in adherence and in clinical outcomes in 11 of 14 included studies[28]. However, most of the included studies were of poor methodological quality, and only three, in the setting of communicable diseases, examine fixed-dose combination pills.

*Is there a potential role for fixed-dose combination therapy in reducing the treatment gap for patients at high risk of CVD? *To address this question, a randomised controlled trial will be conducted to determine whether a polypill-based strategy for both Indigenous and non-Indigenous peoples at high risk of a cardiovascular event will result in better adherence to indicated therapies, and thus lower blood pressure and serum cholesterol levels, when compared with usual care. This will be supplemented by an economic evaluation of the cost-effectiveness of this strategy within partner Aboriginal Medical Services and mainstream General Practices and by a process evaluation to explore the underlying reasons why this strategy might, or might not, be more effective than standard care in different settings.

## Methods & Design

**Figure 1:**
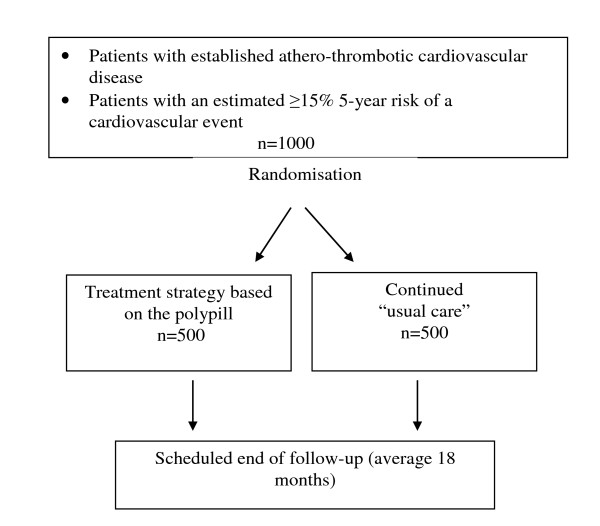
**Study Schema**.

This study is a prospective, open, randomised clinical trial of a polypill-based strategy compared with usual care among Indigenous and non-Indigenous Australians at high CVD risk (Figure 1).

### Subjects

The trial will include 1000 participants with either a previous history of athero-thrombotic vascular disease or who have a 5-year calculated CVD risk of 15% or higher. Patients will be recruited from KVC partner Aboriginal Medical Services (AMS) partners and mainstream general practices. Participants will be eligible for the trial if they are aged 18 years or over and able to give informed consent, have a history of coronary heart disease (myocardial infarction, stable or unstable angina pectoris, or coronary revascularization procedure), and/or ischaemic cerebrovascular disease, and/or peripheral vascular disease; or a calculated 5-year CVD risk of 15% or greater. Five-year CVD risk will be calculated using the 1991 Anderson Framingham risk equation[29]. Adjustments to the estimated risk defined by the New Zealand Guideline Group recommendations, including a 5% increase for being of Indigenous background, will be made[5]. For a participant to be eligible, the responsible medical practitioner should believe that each of the polypill components are indicated and could be prescribed under the Pharmaceuticals Benefit Scheme.

Participants will be ineligible for the trial if there is a contraindication to any of the components of the polypill or if the responsible clinician feels that a change in current therapy will place the patient at risk. Final decisions about eligibility will be made by the clinician and the potential study participant.

### Randomisation

This will be conducted through a central, computer-based randomisation service, and will be stratified by study centre, indication (i.e. previous CVD vs. ≥15% 5-year CVD risk), and prescription of all appropriate therapies at baseline (yes vs. no).

### Study Treatment

Participants will be randomised to either usual care or to a polypill-based strategy. For participants randomised to continue their usual care, management will be at the discretion of their General Practitioner, who will be encouraged to provide care consistent with current guidelines. For those randomised to the polypill-based strategy, the participant will be prescribed one of two polypill formulations, at the discretion of the treating clinician, to be taken orally once daily. Each version contains two blood-pressure lowering drugs, a statin and low-dose aspirin. Suggested indications for each formulation are shown in Table 1. The polypill formulations used in this study have been developed and provided free of charge by Dr Reddy's Laboratories, Hyderabad, India.

**Table 1:** Formulations of polypill available for prescription, and recommended indications

Clinical history	Intervention group
Coronary heart disease	Formulation 1*

Cerebrovascular disease	Formulation 2**

Both or other athero-thrombotic vascular disease	Either Formulation 1* or 2**

>15% 5-year CVD risk, but without established CVD	Formulation 2**

*Polypill with aspirin 75 mg, simvastatin 40 mg, lisinopril 10 mg, atenolol 50 mg

**Polypill with aspirin 75 mg, simvastatin 40 mg, lisinopril 10 mg, hydrochlorothiazide 12.5 mg

For patients starting these medications for the first time, the clinician will be able to titrate with low doses of medications that make up the polypill. For patients currently taking existing drugs of these classes, medications will be reduced or discontinued, aiming to maintain existing levels of treatment. During follow-up, use of 'add on' therapy in patients randomised to the polypill-based strategy is unrestricted, at the discretion of the clinician.

In this "real world" implementation trial, the provision of the study treatment aims to mimic the normal prescribing and dispensing environment. Participants randomised to the polypill-based strategy will obtain their study drug in accordance with the standard prescribing and dispensing procedures at each health service. This will vary and might be dispensed on-site (especially at remote clinics) or at a pharmacy. Participating pharmacies will be registered and will dispense the polypill on receipt of a prescription from an authorised GP investigator. As treatment cost might be an important determinant of adherence, it is important that out-of-pocket expenses for the polypill accord with the pricing environment for accessing medications in each health service. Participants randomized to the polypill will pay the same copayment for a polypill script as they would for any other medication. Participants randomised to usual care will continue to fill prescriptions for cardiovascular medications according to their usual custom.

### Follow Up

The average follow-up period will be 18 months. Because this is a pragmatic study evaluating strategies of care (rather than therapeutic agents), an open design is necessary. Appropriate care will be taken to ensure standardised, and where feasible, blinded, outcome evaluation -- "PROBE" design[30]. During follow-up, all study participants will attend clinic visits for randomisation, at 12 months, 24 months (if applicable) and at the end of the study. Research staff will telephone or visit study participants (especially in remote settings) at 6 months and 18 months. All other routine visits to the general practitioner will be at the discretion of that clinician and the patient.

### Outcomes

The primary study outcomes will be changes, from baseline measures, in serum cholesterol and systolic blood pressure and self-reported current use of aspirin, a statin and at least two blood pressure lowering drugs. This will capture the combined effect of changes by the health-care provider (prescription) and by the patient (adherence). The use of biological markers (i.e. levels of blood pressure and cholesterol) directly measure the consequences of changes in adherence, and translate directly to likely improvements in hard clinical outcomes. The secondary outcomes of the trial include cardiovascular events, renal outcomes, self-reported barriers to indicated therapy, prescription of indicated therapy, occurrence of serious adverse events, and quality-of-life.

### Statistical considerations

Assumptions regarding differences in adherence to guideline-indicated therapies are based on evidence from the recent audits of CVD management in both Indigenous and mainstream primary care settings[6-8]. The following assumptions inform sample size estimates: (i) there is 35% baseline adherence to all components of the polypill (i.e. aspirin, statin and at least 2 blood pressure-lowering agents); (ii) adherence to all components of the polypill among participants randomised to "usual care" over the duration of the trial will increase to 50%; and (iii) 80% of participants randomised to the polypill-based strategy will remain adherent to the polypill during follow-up (allowing for 20% drop-out from therapy during follow-up).

Allowing for 10% deaths and loss to follow-up, randomisation of 1000 participants will provide 90% power at a two-sided 0.05 significance level to detect at least a 3 mmHg difference in systolic blood pressure, and at least 0.20 mmol/L difference in serum cholesterol. This assumes that the standard deviations around the baselines for these variables are 14 mmHg and 0.9 mmol/L respectively. This study is underpowered to detect plausible differences in cardiovascular events, but the data from Kanyini GAP will contribute to a prospective meta-analysis including other international polypill trials with similar protocols. All analyses will be performed on an intention-to-treat basis. An independent Data and Safety Monitoring Committee will be established to review unblinded interim data on efficacy and safety.

### Economic evaluation of the Polypill-based strategy

A cost-effectiveness analysis, taking a health system perspective, will compare the polypill-based strategy with usual care. This will entail a trial-based economic evaluation and a modelled economic evaluation of long-term costs and outcomes. In the *trial based economic evaluation*, the costs of medications, based on actual market prices for each item and a range of indicative prices for the polypill, will be compared between the two groups (including follow-up of patients who fail to adhere to allocated treatment). Costs of other medications, laboratory tests and medical consultations will be extracted from Medicare and Pharmaceutical Benefits Scheme (PBS) records and costed at prevailing rates. Hospitalisations will be recorded routinely as serious adverse events in the trial and will be costed using standard Australian National Diagnosis Related Groups (AN-DRG) cost weights. In addition, the measures of self-reported health based on the EQ 5 D administered at baseline, 12 months and the final visit will enable estimates of quality of life[31]. Given the emphasis on promoting access to care, out-of-pocket costs will be compared between the two groups as a separate analysis. These will include patient co-payments associated with medications and with medical consultations. The trial-based economic evaluation will estimate the incremental cost consequences of the polypill strategy in achieving each of the primary outcomes and will estimate the incremental cost per Quality Adjusted Life Year (QALY) at 18 months.

A *modelled economic evaluation *will be done, using a state transition or Markov model, to capture costs and outcomes which occur beyond the period of the trial. This will enable quality of life and survival to be examined beyond the 18-month follow-up. Using the Markov model, patients in usual care and the polypill-based strategy would be tracked over an extended period to capture various health states. Transition from good health to major morbidity (for example stroke), or mortality, will be based on probabilities related to the long-term effects of blood pressure and cholesterol lowering, and anti-platelet therapy, medication safety and disease progression, derived from the trial findings and literature review. Costs and quality of life attached to various states of health will be based on findings from the trial. With appropriate discounting, estimates of long-term costs and outcomes will be derived from the model. Sensitivity analyses will be conducted on the discount rate, uncertainty in outcome estimates and assumptions made in the costings. In addition, given that the market price for the polypill is yet to be established, different pricing scenarios will be tested to determine the threshold values for achieving cost-effectiveness.

### Process evaluation of the Polypill-based Strategy

A process evaluation will explore the barriers and enablers to implementing a polypill-based strategy to enhance prescriber and consumer adherence to the indicated therapies[32]. This will inform the interpretation of the key findings of the trial, considerations regarding transferability of the results to other settings, and will assist in translating findings into policy and practice[33]. Mid-way through follow-up, semi-structured interviews will be conducted with key prescribers and other staff in participating AMSs and mainstream general practices. We aim to explore their views on the advantages, disadvantages, acceptability and applicability of the polypill strategy along with their accounts of how participation in the study itself changed their prescribing behaviour. At the end of follow-up, selected patients will be interviewed to explore their views on the benefits, disadvantages and acceptability of the polypill. Recruitment of staff and patients for interviews will be purposive, to maximise variation according to criteria including location, service size, role and degree of participation (for staff); and location, sex, age and outcomes (for patients). Analysis of the interview data will be primarily thematic[34] and will be informed by the realistic evaluation model of Pawson and Tilley[35], which seeks to understand human choices, actions and attitudes, within the context of the systems in which these players operate.

A multi-disciplinary team, comprised of Indigenous and non-Indigenous, clinical and non-clinical research staff, will undertake the analysis to ensure that its interpretation is sensitive to different perspectives. Using the constant comparative method [36], analyses will occur concurrently with interviews and themes will be continually modified by the team in the light of additional data. NVivo (QSR International, Melbourne, Victoria) will be used to assist with data management. This software is particularly useful when there are multiple coders across several sites, allowing us to bring local, context-rich analyses to interpretation of the findings.

### Study organisation and Timelines

The study will be centrally managed by The George Institute, with the design and conduct overseen by a Steering Committee. Regional management of study centres will be the responsibility of the George Institute (Sydney-based), the Baker IDI Centre for Indigenous Vascular & Diabetes Research (Alice Springs-based) and Monash University (Melbourne-based). Ethics approval was first granted by Sydney South West Area Health Service Human Research Ethics Committee (HREC) (RPAH zone), and subsequently by relevant HRECs across the country which are the Aboriginal Health & Medical Research Council HREC, Cairns and Hinterland Health Service District HREC, Central Australian HREC, Metro South Health Service District HREC and Monash University HREC. Participant recruitment commenced in December 2009 and should be completed by July 2011. Follow-up will continue until December 2012. Process evaluation interviews will occur mid-trial and at the end of follow-up. The economic evaluation, including data collection and modelling, will occur throughout. Publication of final results is planned for early 2013.

## Discussion

This clinical trial is one study within the KVC health services research program. In 2006, with funding from a national, competitive, peer-reviewed grant, KVC aimed to explore barriers and facilitators to the delivery of high quality care and to develop culturally appropriate, innovative and accessible models of care. KVC's achievements include an audit of the detection, prevention and management of chronic vascular diseases[7] and a qualitative study exploring barriers to improving health service delivery. The Kanyini-GAP polypill study brings together researchers, health service providers and research institutes across Indigenous and mainstream health sectors to undertake a clinical trial, economic and process evaluations. We believe that working across this apparent health sector divide, will strengthen our ability to change health service provision to improve population health.

This clinical trial component has an ambitious recruitment period of 18 months, which might be a challenge for both AMSs and mainstream primary care settings. Reasons include a generally negative perception of research in many Indigenous communities[37-39], the considerable length of time required for consultation with communities to form research partnerships[38,40] and the practicalities of conducting clinical trials in busy AMSs and primary health care settings within the limitations of non-commercial research funding[41,42]. The initial consultations with partner AMSs have set the research agenda and the strength of our partnerships will be fundamental to successfully implementing the trial across remote, rural and urban settings within the intended time-frame.

This clinical trial is consistent with KVC's core objective to build the capacity of community members and health services to conduct rigorous, high-quality research and to develop, implement and evaluate interventions to address barriers to care. The research capacity of the Indigenous workforce is enhanced through the employment, support and training of Indigenous Research Fellows (IRFs) based at our partner AMSs. KVC directly supports the IRFs in their conduct of the study through regular face-to-face and teleconference meetings, supports attendance at research courses and encourages the use of research findings to satisfy post-graduate course requirements. This support, provided in collaboration with each IRF's employing health service, has sustained the development of Indigenous researchers within a culturally and academically safe environment. KVC has benefitted from this reciprocal capacity-building process, whereby Indigenous attitudes and community priorities have been promoted to non-Indigenous researchers to ensure appropriate cross-cultural communication and accountability in the analysis and dissemination of findings.

The Kanyini-GAP study team aims to generate robust evidence to persuade governments to change policy. Having demonstrated systemic evidence-practice gaps across AMS and mainstream primary care sectors, we believe a polypill-based strategy for people at high cardiovascular risk might address these gaps, especially amongst disadvantaged populations. In addition, through partnership with AMS and mainstream primary care services, we aim for greater generalisability of trial results. In this manner we hope to contribute to sustained improvements in health for all Australians.

## Competing interests

Anushka Patel, David Peiris and Alan Cass received reimbursements for travel and accommodation funded by Doctor Reddy's Laboratory, Hyderbad, India to attend international Polypill collaborative meetings. Other author(s) declare that they have no competing interests.

## Authors' contributions

The writing group of this manuscript are members of the Kanyini Vascular Collaboration and Kanyini GAP study team. Different members of the writing group have contributed significantly to writing and manuscript revision, study design, securing of funding, stake holder engagement, ethics approval and oversight of study implementation and the study implementation at sites. The writing group comprise of HL, AP, AB, SE, NH, SJ, IR, GS, AT, TW, VW, AR, TU, BN, DP, HB, CR and AC. All authors read and approved the final manuscript.

## Pre-publication history

The pre-publication history for this paper can be accessed here:

http://www.biomedcentral.com/1471-2458/10/458/prepub
